# Quercitrin Attenuates Acetaminophen-Induced Acute Liver Injury by Maintaining Mitochondrial Complex I Activity

**DOI:** 10.3389/fphar.2021.586010

**Published:** 2021-05-05

**Authors:** Weichen Xiong, Zixin Yuan, Tianshun Wang, Songtao Wu, Yiyi Xiong, Yunfeng Yao, Yanfang Yang, Hezhen Wu

**Affiliations:** ^1^ Faculty of Pharmacy, Hubei University of Chinese Medicine, Wuhan, China; ^2^ Key Laboratory of Traditional Chinese Medicine Resources and Chemistry of Hubei Province, Wuhan, China; ^3^ Collaborative Innovation Center of Traditional Chinese Medicine of New Products for Geriatrics Hubei Province, Wuhan, China; ^4^ Key Laboratory of Traditional Chinese Medicine Resource and Compound Preparation Ministry of Education, Hubei University of Chinese Medicine, Wuhan, China

**Keywords:** quercitrin, acute liver injury, acetaminophen, complex I, mitochondrial dysfunction, reactive oxygen species

## Abstract

The flavonoid quercitrin has a strong antioxidant property. It is also reported to have a protective effect on the liver. However, the mechanism by which it exerts a protective effect on the liver is not fully understood. The objective of this article is to confirm the protective effect of quercitrin extracted from Albiziae flos on acetaminophen (APAP)-induced liver injury and to explain its mechanism. In the *in vivo* study, quercitrin was administered orally to BALB/c mice at a dose of 50, 100, and 200 mg/kg for seven consecutive days. APAP (300 mg/kg) was injected intraperitoneally after a last dose of quercitrin was administered. Determination of alanine aminotransferase (ALT), aspartate aminotransferase (AST), lactate dehydrogenase (LDH), interleukin 6 (IL-6), tumor necrosis factor α (TNF-α), reactive oxygen species (ROS), superoxide dismutase (SOD), glutathione (GSH), glutathione peroxidase (GSH-Px), catalase (CAT), and malondialdehyde (MDA) levels showed that quercitrin effectively attenuated APAP-induced acute liver injury in mice. Results of the *in vitro* study showed that quercitrin reduced the levels of ROS, protected mitochondria from damage, and restored the activity of mitochondrial complex I in APAP-treated L-02 cells. The addition of rotenone which is an inhibitor of complex I blocked the protective effect of quercitrin. The expression of mitochondrial complex I was also maintained by quercitrin. Our results suggest that quercitrin can maintain the level of mitochondrial complex I in injured cells and restore its activity, which reduces the production of ROS, protects the mitochondria from oxidative stress, and has a protective effect on the liver.

## Introduction

The liver which is one of the important organs in the human body is responsible for metabolizing ingested compounds and removing toxic substances (Mitra and Metcalf, 2012). Injury to the liver can be caused by hepatotoxic compounds like acetaminophen (APAP), for example. APAP is widely used clinically as an antipyretic and an analgesic drug (Lee, 2017). Although it is safe and effective at therapeutic doses, higher doses of APAP are hepatotoxic and can cause acute liver injury. Abuse of APAP is currently the main cause for failure of the liver (Yan et al., 2018). N-acetylcysteine (NAC) is currently the only U.S. Food and Drug Administration–approved antidote for treating toxicity of APAP. However, long-term use of NAC can cause toxic side effects and damage regeneration of the liver (Yang et al., 2009). Hence, newer alternative drugs are urgently needed to treat APAP-induced acute liver injury.

Numerous studies have shown that excessive APAP exposure can lead to mitochondrial dysfunction, increased ATP consumption, and increased generation of reactive oxygen species (ROS) and oxidative stress (Subramanya et al., 2018). All of these factors ultimately result in injury to the liver (Masubuchi et al., 2005; Jaeschke and Bajt, 2006). Respiration is accomplished by the electron transport chain (ETC) located on the mitochondrial inner membrane, and the mitochondrial complex I is an important component of the ETC (Guo et al., 2018). Reduction in the activity of complex I results in electron leakage, which in turn leads to the generation of ROS and mitochondrial dysfunction (Dröse et al., 2016). The activity of mitochondrial complex I is regulated by the mitochondrial negative regulatory protein methylation–controlled J (MCJ) protein (Hatle et al., 2013). A previously published study has reported that excessive APAP can increase the expression of MCJ in mice (Barbier-Torres et al., 2017). MCJ binds to mitochondrial complex I, prevents the formation of supercomplexes, and inhibits the activity of complex I. This results in an increased production of mitochondrial ROS and oxidative stress (Ramachandran et al., 2018). Restoring the activity of mitochondrial complex I is therefore one of the potential targets for liver protection.

Flavonoids are a group of natural substances with variable phenolic structures (Panche et al., 2016). Studies have reported that flavonoids reduced APAP-induced liver injury due to their powerful antioxidant effect (Kuang et al., 2017; Li et al., 2017; Shao et al., 2017; Chowdhury et al., 2019). Therefore, we have investigated the protective effect of Albiziae flos (dry inflorescence or the bud of *Albizia julibrissin* Durazz.), a traditional Chinese medicine rich in flavonoids, on APAP-induced acute liver injury and found that its active ingredient is quercitrin (Que). As a natural flavonoid with a wide range of pharmacological activities, quercitrin has been proven to have antioxidant, anti-inflammatory, antitumor, and antidepressant effects (Camuesco et al., 2004; Li et al., 2016; Xiong et al., 2020). Quercitrin is known to have protective effects on liver injury; however, its mechanism of action remains to be further elucidated (Raja et al., 2007; Truong et al., 2016).

In this study, we established the efficacy of quercitrin in attenuating APAP-induced acute liver injury and studied its effect on complex I to further clarify its mechanism of action at a molecular level.

## Materials and Methods

### Chemicals and Reagents

Albiziae flos was purchased from Shizhentang Badong Pharmaceutical (Lot: 19021502, Enshi, China). APAP, silymarin (Sil), and rotenone were purchased from Macklin (Shanghai, China). The hematoxylin and eosin (H & E) dye kit was purchased from Baiqiandu (Wuhan, China). Alanine aminotransferase (ALT), aspartate aminotransferase (AST), lactate dehydrogenase (LDH), superoxide dismutase (SOD), glutathione (GSH), glutathione peroxidase (GSH-Px), catalase (CAT), and malondialdehyde (MDA) commercial kits were purchased from Nanjing Jiancheng Bioengineering Institute (Nanjing, China). The BCA protein concentration assay kit was purchased from Biosharp (Hefei, China). Dihydroethidium (DHE), 4′,6-diamidino-2-phenylindole (DAPI), and RIPA lysis buffer were purchased from Beyotime (Shanghai, China). Interleukin 6 (IL-6) and tumor necrosis factor α (TNF-α) ELISA kits were purchased from Elabscience (Wuhan, China). The electron transport chain complex I assay kit and mitochondrial protein extraction kit were purchased from Nanjing Jiancheng Bioengineering Institute (Nanjing, China). DCFH-DA and JC-1 were purchased from Solarbio (Beijing, China). F4/80, apoptosis-inducing factor (AIF), endonuclease G (EndoG), NDUFS3, and GAPDH antibodies were purchased from Proteintech (Rosemont, IL, United States). The anti-rabbit secondary antibody was purchased from Cell Signaling Technology (Danvers, MA, United States). The ELC chemiluminescence kit and nitrocellulose (NC) membrane were purchased from Bio-Rad (Hercules, CA, United States). HPLC grade solvents were purchased from Tedia (Fairfield, OH, United States). All other solvents were of analytical grade (Sinopharm Chemical Reagent, Shanghai, China).

### Preparation of Quercitrin From Albiziae Flos

The powder of Albiziae flos (10 kg) was extracted with 80% EtOH (100 L). The combined extracts were concentrated by a rotary evaporator. The concentrate was successively extracted with petroleum ether, ethyl acetate, and n-butanol. The ethyl acetate extract was further separated on the silica gel column (100–200 mesh), and three subfractions were obtained by gradient elution with trichloromethane-methanol (6:1–2:1). The solvent of subfraction 2 was recovered, and the remaining extract was fully mixed with trichloromethane–methanol (1:1) solution and then filtered after standing overnight. The filtrate residue was dried, and the yellow powder was obtained as quercitrin. The purity was detected by a 1260 high-performance liquid chromatograph (HPLC) (Agilent, CA, United States), and the structure was analyzed by a Xevo G2-XS QTOF mass spectrometer (Waters, MA, United States) and Bruker Avance III 800 MHz instrument (Bruker, Karlsruhe, Germany).

### Animals and Treatments

Male BALB/c mice of 18–22 g body weight were provided by the Hubei Provincial Center for Disease Control and Prevention. The mice were kept at a temperature of 24 ± 2°C and a humidity of 50 ± 5% and were exposed to 12 h of light and 12 h of darkness each day. All animals had free access to food and water. All animal protocols were approved by the Experimental Animal Ethics Committee of the Hubei University of Chinese Medicine (NO. HUCMS202006011).

Following one week of adaptive feeding, 60 mice were randomly separated into following six groups (*n* = 10): a control group, a model group (APAP group), three treatment groups (APAP +50 mg/kg Que group; APAP +100 mg/kg Que group; APAP +200 mg/kg Que group), and a positive control group (APAP +100 mg/kg Sil group).

APAP (300 mg/kg) was dissolved in sterile physiological saline; quercitrin and silymarin were formulated as a suspension with 0.5% (w/v) CMC-Na. Mice in the three treatment groups were orally administered with quercitrin suspension for seven consecutive days, and a single APAP (300 mg/kg) was injected intraperitoneally after 1 h of the last administration of quercitrin. The positive control group was administered in the similar manner. Control group and APAP group mice were orally administered with 0.5% CMC-Na solution for seven consecutive days. One hour after the last administration of CMC-Na solution, mice in the control group were intraperitoneally injected with saline, whereas mice in the APAP group were intraperitoneally injected with a single dose of APAP (300 mg/kg). The volume of oral administration and intraperitoneal injection was 0.1 ml/10 g.

In order to eliminate the effect of diet on APAP modeling, all mice were not given any food for 15 h before intraperitoneal injection and were given food following the intraperitoneal injection. All mice were sacrificed after they were treated with APAP for 14 h, and their blood samples and liver tissues were collected for further analysis. The blood samples were centrifuged (4°C, 4000 rpm, and 10 min) to separate the serum and stored at −20°C. After weighing the liver tissues, one part was fixed in 4% paraformaldehyde and the other was stored at −80°C.

### Histochemical Staining

The liver tissues were fixed with 4% paraformaldehyde for 24 h and then embedded in paraffin. Next, the tissues were cut into 5-μm sections. Hematoxylin and eosin (H&E) were used for staining, and the F4/80 antibody was used for immunohistochemical staining.

To determine the levels of ROS, the frozen liver tissues were cut into 5-μm sections, stained with DHE, and then counterstained with DAPI. The sections were examined under the Nikon Eclipse C1 fluorescence microscope imaging system (Nikon Instruments, Tokyo, Japan).

### Determination of Serum ALT, AST, and LDH

ALT and AST were determined according to the following steps. In short, 5 μL of diluted serum was incubated with 20 μL of ALT/AST matrix solution at 37°C for 30 min, and then, 20 μL of 2,4-dinitrophenylhydrazine hydrochloride solution was added to incubate at 37°C for another 20 min. Finally, 200 μL 0.4 M NaOH was added to stop the reaction. After 15 min at room temperature, absorbance at 510 nm was read by a SPARK 10 M microplate reader (Tecan, Switzerland), and the ALT/AST activity was calculated according to the standard curve. The LDH activity was determined according to the manufacturer’s instructions.

### Determination of Inflammatory Factors

The levels of IL-6 and TNF-α in the serum were determined according to the instructions provided by the manufacturers of ELISA kits. The absorbance at 450 nm was measured using a SPARK 10 M microplate reader (Tecan, Switzerland).

### Determination of Hepatic SOD, GSH, GSH-Px, CAT, and MDA

Appropriate amount of thawed frozen liver tissue was taken, and 9-fold volume of 4°C physiological saline was added to homogenize and centrifuged (4°C, 4000 rpm and 10 min). Following centrifugation, the supernatant was collected. Protein concentrations in liver homogenates were measured using the BCA protein concentration assay kit. The SOD, GSH, GSH-Px, CAT, and MDA content in the liver homogenates were determined according to the instructions provided by the respective assay kits.

### Cell Culture

The L-02 (HL-0072) cell line was obtained from the Key Laboratory of Traditional Chinese Medicine Resources and Chemistry of Hubei Province (Wuhan, China). L-02 cells were cultured with RPMI-1640 medium comprising 10% fetal bovine serum (FBS) in a 5% CO_2_ incubator at 37°C and humid atmosphere.

### Cell Viability Assay

L-02 cells (1 × 10^5^ cells/well) were sowed in 96-well plates and divided into five groups: a control group, a model group (APAP group), and three treatment groups. The cells were cultured overnight, and after checking for cell adherence, the cells in the treatment groups were treated with quercitrin at final concentrations of 20, 40, and 80 μM for 15 min. Next, APAP (10 mM) was added to each group, except the control group. After 24 h, each group was incubated with MTT (5 mg/mL) for 4 h. Following this, the supernatant was removed carefully, and 150 μL of DMSO was added to each well to completely dissolve the purple crystals of formazan. The optical density (OD) value at 490 nm was measured by using a microplate reader. In order to determine the effect of rotenone, a complex I inhibitor, on quercitrin activity, rotenone (10 μM) was added into the wells after 5 h of treatment by APAP.

### Determination of ROS and Mitochondrial Membrane Potential

L-02 cells (2.5 × 10^5^ cells/well) were seeded in 6-well plates and divided into four groups: a control group, a drug group [Que group, cells were treated with quercitrin (80 μM) only], a model group [APAP group, cells were treated with APAP (10 mM) only], and a treatment group (APAP + Que group, cells were incubated with quercitrin for 15 min and then treated with APAP). After 24 h incubation, ROS was determined by flow cytometry (FACSCalibur, BD Company, NJ, United States) after staining with DCFH-DA. The mitochondrial membrane potential was observed with a fluorescence microscope (Nikon Instruments, Tokyo, Japan) after staining with JC-1.

### Determination of Complex I Activity

Total mitochondrial proteins of L-02 cells were extracted by using a mitochondrial protein extraction kit. Complex I catalyzes the conversion of reduced nicotinamide adenine dinucleotide (NADH) to nicotinamide adenine dinucleotide (NAD). Therefore, the activity of complex I was calculated by measuring the decrease in absorbance at 340 nm to determine the conversion rate of NADH according to the manufacturer’s instructions.

### Western Blot Analysis

Total protein extracts of cells were prepared using RIPA lysis buffer. Protein concentrations were determined in all samples using the BCA protein concentration assay kit. Protein (equal concentration) samples were separated by running them on a 10% SDS-PAGE gel and transferred onto an NC membrane. This NC membrane was blocked using 5% (w/v) nonfat milk at room temperature for 2 h. Following blocking, the membrane was incubated with the primary antibody at 4°C overnight. The next day, the membrane was incubated with the anti-rabbit secondary antibody for 2 h at room temperature. The protein bands were detected by chemiluminescence using the ECL reagent and photographed using a FluorChem FC3 gel imager.

### Statistical Analysis

All data are presented as mean ± standard deviation (SD). A one-way analysis of variance (ANOVA) was used for data analysis. *p* < 0.05 or *p* < 0.01 represented statistically significant difference.

## Results

### Identification of Quercitrin From Albiziae Flos

**FIGURE 1:**
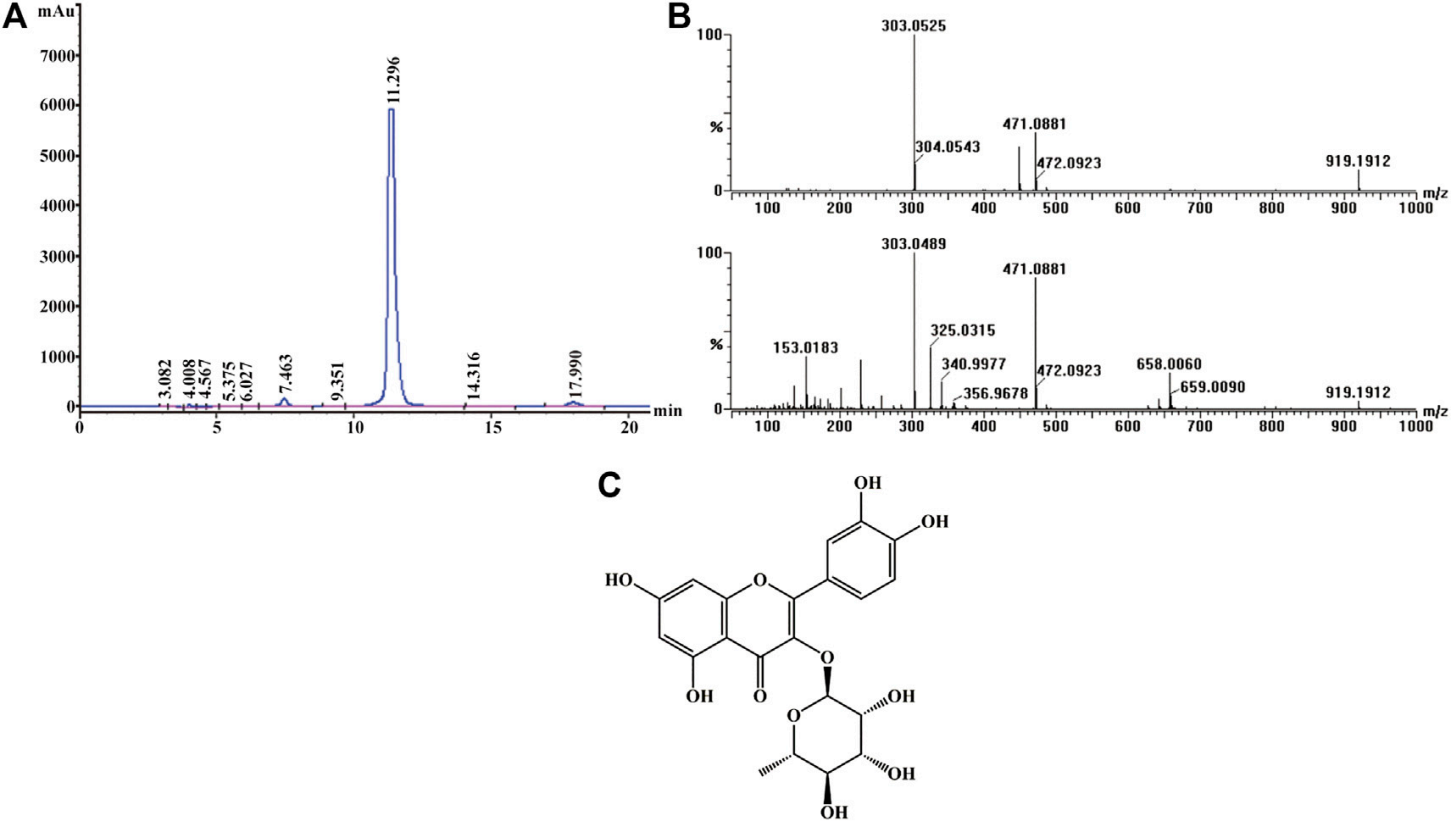
Structural identification of quercitrin. **(A)** HPLC-UV chromatogram obtained at 210 nm. **(B)** MS (top) and MS/MS (bottom) data for quercitrin. **(C)** Structural formula of quercitrin.

Active screening showed that quercitrin was the main active ingredient in Albiziae flos, so quercitrin was extracted for subsequent experiments (Supplementary Figures S1, S2; Supplementary Table S1). The obtained yellow powder was easily soluble in methanol, and its purity was more than 95% by HPLC (Figure 1A). Mass spectrometry showed that its HR-ESI-MS *m/z* 471.0881 ([M + Na]^+^, calcd. 471.0903) and *m/z* 449.1083 ([M + H]^+^, calcd. 449.1084), which indicated that the molecular formula of this compound was C_21_H_20_O_11_ (Figure 1B). Finally, ^1^H- and ^13^C-nuclear magnetic resonance (NMR) analysis showed that the yellow powder was quercitrin (Supplementary Table S2, Figure 1C).

### Quercitrin Attenuates APAP-Induced Acute Liver Injury in Mice

**FIGURE 2:**
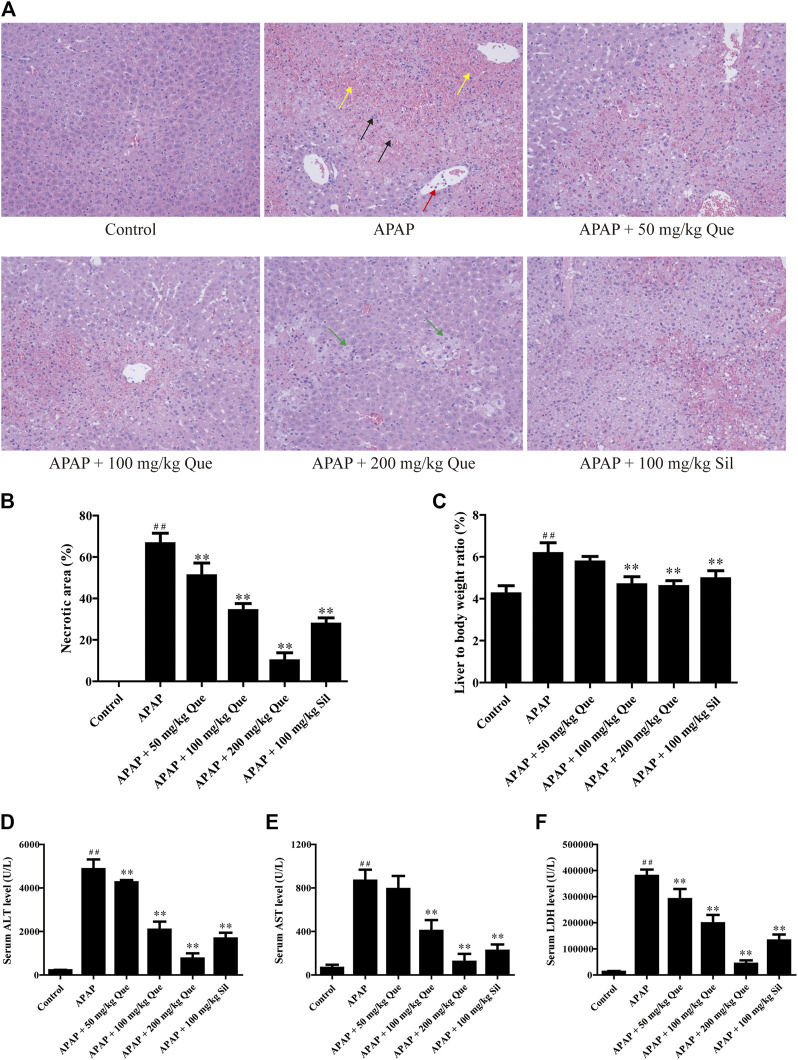
Effect of quercitrin on APAP-induced acute liver injury in mice. **(A)** Pathological evaluation of the liver tissue of mice (H & E staining 200×). Black arrows show cell nuclei fragmentation; yellow arrows show hepatic sinusoidal congestion; red arrow shows neutrophil aggregation; green arrows show hepatocyte edema. **(B)** Necrotic area of the liver tissue. **(C)** Effect of quercitrin on liver organ coefficients of mice. **(D–F)** Analysis of ALT, AST, and LDH contents in the serum of mice. The experimental data are presented as mean ± SD, ^##^ *p* < 0.01, compared with the control group; **p < 0.01, compared with the APAP group.

In the beginning, we evaluated the protective effect of quercitrin on the liver. We first did a gross examination of the livers of the mice. Histopathological results are presented in Figure 2A. Compared with the control group, the liver cells in the APAP group were necrotic in a great area, as indicated by the black arrow; the necrotic area was filled with red blood cells, as indicated by the yellow arrow; and neutrophils were aggregated, as indicated by the red arrow. Moreover, the cellular structure of the liver tissue in quercitrin-pretreated mice tended to be normal compared with the APAP group. In these quercitrin-pretreated mice, the inflammatory infiltration was significantly decreased, and only a few hepatocytes were edema, as indicated by green arrows. The necrotic area of the liver tissue was determined. Massive necrotic areas occurred in the APAP group. In contrast, quercitrin pretreatment effectively reduced liver cell necrosis in a dose-dependent manner (Figure 2B). Furthermore, the liver organ coefficients were calculated. The liver organ coefficient of the APAP group was significantly augmented compared with the control group (*p* < 0.01). Quercitrin 100 and 200 mg/kg pretreatment significantly decreased the organ coefficient of the liver (*p* < 0.01) (Figure 2C).

There was a significant increase in serum levels of ALT, AST, and LDH in the APAP group compared with the control group (*p* < 0.01). Furthermore, quercitrin pretreatment significantly reduced ALT, AST, and LDH in serum compared with the APAP group (*p* < 0.01) and exhibited a dose-dependent effect (Figures 2D–F). The abovementioned findings indicated that quercitrin could diminish APAP-induced acute liver injury in mice and improve APAP-induced liver enlargement and cell injury.

### Quercitrin Reduces APAP-Induced Inflammation

**FIGURE 3:**
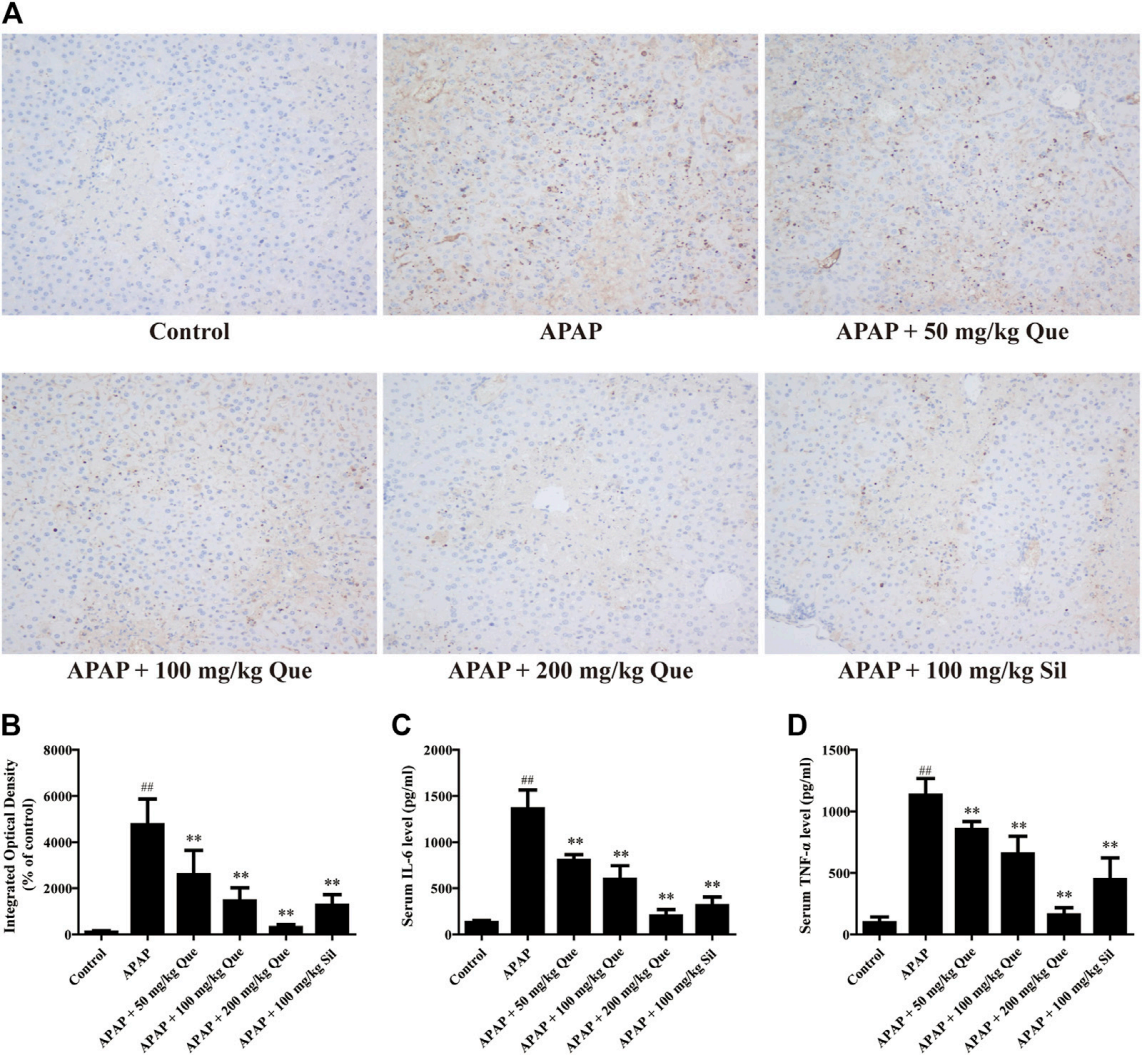
Effect of quercitrin on APAP-induced inflammation. **(A)** Effect of quercitrin on F4/80 expression in the liver tissue of mice (200×). **(B)** IOD of immunohistochemistry. **(C,D)** Effect of quercitrin on serum IL-6 and TNF-α levels in APAP-induced liver injury mice. The experimental data are expressed as mean ± SD, ^##^ *p* < 0.01, compared with the control group; **p < 0.01, compared with the APAP group.

In order to further confirm that quercitrin has a protective effect on the liver, the levels of inflammatory factors were determined. Results of the F4/80 protein content are shown in Figure 3A, and the brown part is the expression of F4/80. The sections were analyzed by integrated optical density (IOD), and the results were consistent with those observed in the sections (Figure 3B). F4/80 expression in the APAP group increased significantly when compared with the control group (*p* < 0.01). Pretreatment with quercitrin and silymarin significantly reduced the increase in F4/80 caused by APAP (*p* < 0.01). The above results demonstrated that APAP increased the macrophage content in the liver, which decreased after quercitrin treatment.

Additionally, the serum levels of IL-6 and TNF-α in the APAP group were significantly increased when compared with the control group (*p* < 0.01). Compared with the APAP group, the levels of IL-6 and TNF-α in the serum of mice pretreated with quercitrin were significantly reduced (*p* < 0.01) (Figures 3C,D). The reduction of pro-inflammatory cytokines also indicated that quercitrin can attenuate APAP-induced liver injury.

### Quercitrin Alleviates APAP-Induced Oxidative Stress

**FIGURE 4:**
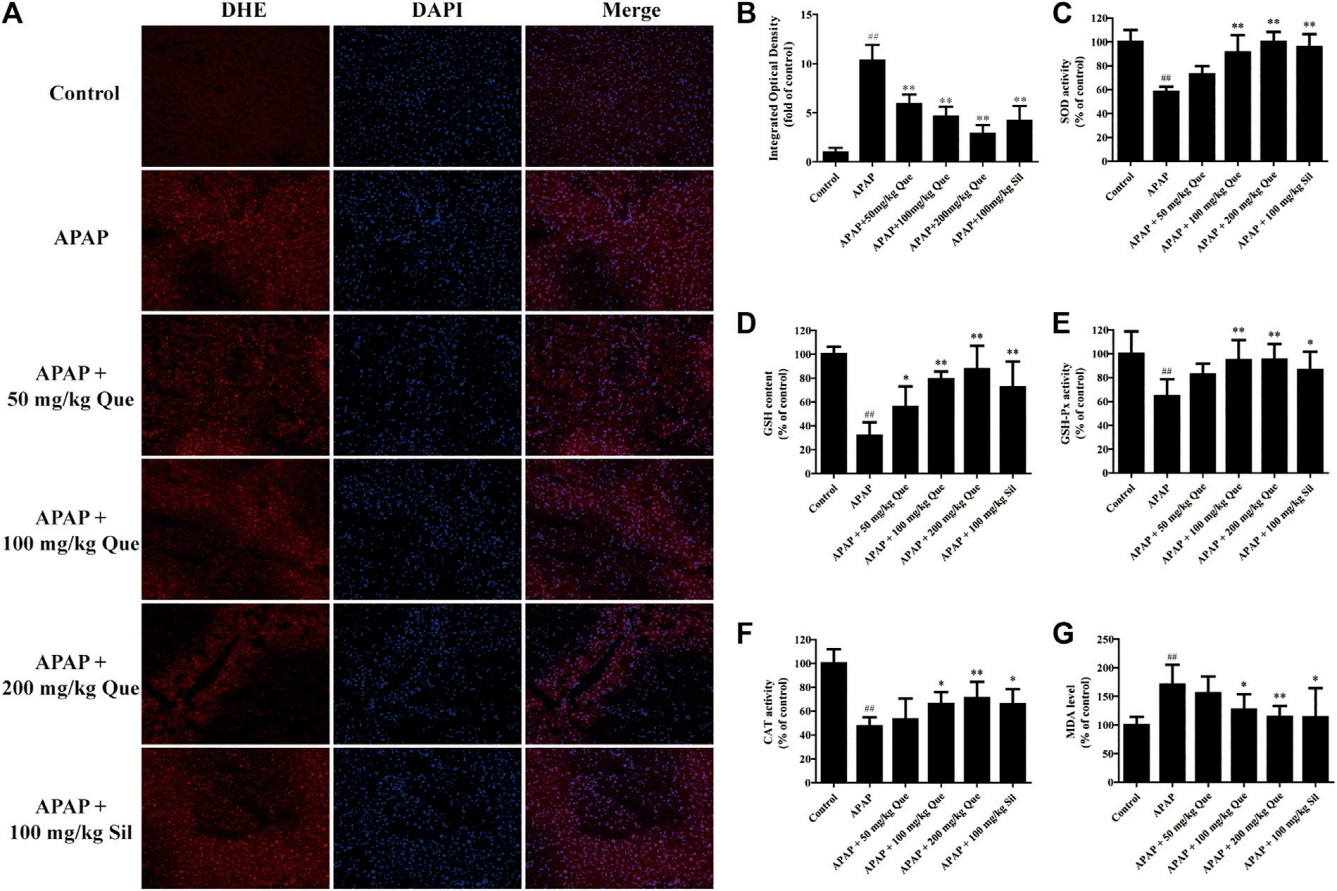
Effect of quercitrin on APAP-induced oxidative stress. **(A)** ROS frozen sections of the liver tissue of mice (DHE staining 200×). **(B)** IOD of frozen sections. **(C–F)** SOD, GSH, GSH-Px, and CAT levels in the liver of mice. **(G)** MDA content in the liver of mice. The experimental data are expressed as mean ± SD, ^##^ *p* < 0.01, compared with the control group; *p < 0.05, **p < 0.01, compared with the APAP group.

Next, the effect of quercitrin on APAP-induced oxidative stress was evaluated. The ROS fluorescent sections were observed, and the sections were analyzed by IOD analysis (Figures 4A,B). There was a significant augmentation of the ROS levels in the APAP group compared with the control group (*p* < 0.01). In addition, there was a significant decrease in the ROS levels in the three treatment groups compared with the APAP group (*p* < 0.01).

The results of the antioxidant enzymes assay showed that SOD, GSH, GSH-Px, and CAT levels in the APAP group were significantly reduced compared with the control group (*p* < 0.01). Pretreatment with quercitrin significantly increased the levels of SOD, GSH, GSH-Px, and CAT (*p* < 0.05, *p* < 0.01) and showed a dose-dependent effect. The efficacy of silymarin was comparable to that of quercitrin at 100 mg/ml (Figures 4C–F). In addition, the content of MDA in the APAP group was significantly increased when compared with the control group (*p* < 0.01). Pretreatment with quercitrin 100 and 200 mg/kg significantly reduced the levels of MDA when compared with the APAP group (*p* < 0.05, *p* < 0.01) (Figure 4G). Above results indicated that quercitrin can alleviate APAP-induced acute liver injury by reducing oxidative stress injury in the liver of mice.

### Quercitrin Increases the Activity and Reduces ROS Levels of APAP Treated L-02 Cells

**FIGURE 5:**
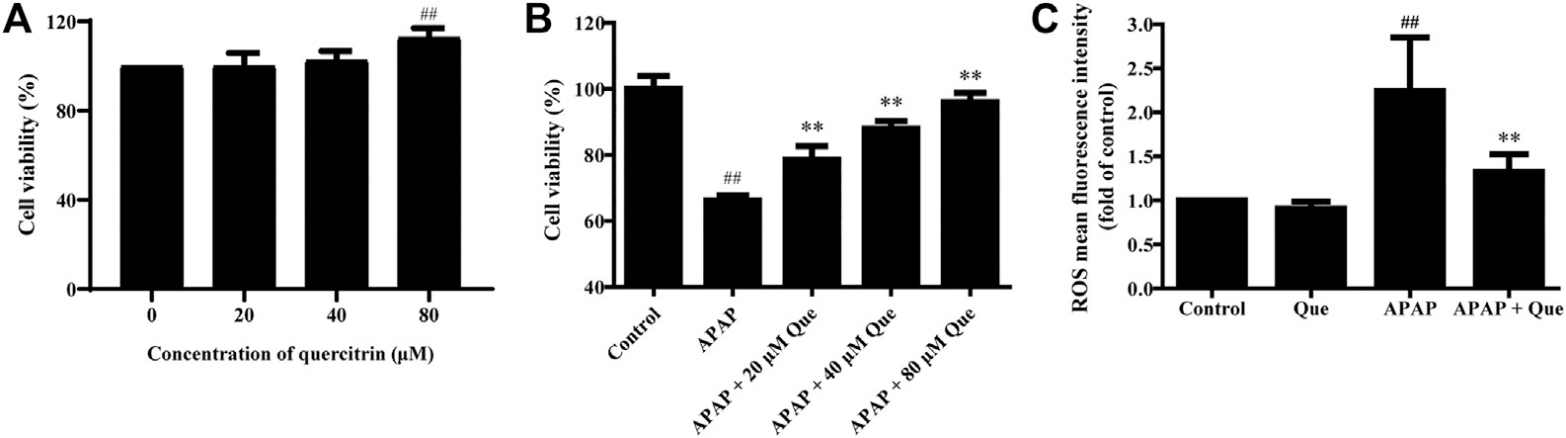
Effect of quercitrin on the activity and ROS levels of APAP-treated L-02 cells. **(A)** Effect of quercitrin on the viability of L-02 cells. **(B)** Effect of quercitrin on the viability of APAP-treated L-02 cells. **(C)** Effect of quercitrin on ROS levels in L-02 cells. The experimental data are expressed as mean ± SD, and the experiment was repeated three times, ^##^ *p* < 0.01, compared with the control group; **p < 0.01, compared with the APAP group.

First, the *in vitro* activity of quercitrin was determined. 80 μM quercitrin significantly increased cell viability, APAP (10 mM) decreased cell viability to 66.26 ± 1.54%, while pretreatment with quercitrin restored cell viability (*p* < 0.01) (Figures 5A,B). These results demonstrated that quercitrin could effectively reduce APAP-induced L-02 cell injury.

Next, we studied the effect of quercitrin on the ROS levels of L-02 cells. There was no significant difference in the levels of ROS between the Que group and the control group. However, the levels of ROS in the APAP group were significantly higher than those in the control group (*p* < 0.01). The ROS levels decreased significantly in the APAP + Que group when compared with the APAP group (*p* < 0.01) (Figure 5C). The above results demonstrated that pretreatment with quercitrin did not affect the ROS levels of normal cells but decreased the ROS levels of L-02 cells which were treated with APAP.

### Quercitrin Prevents Mitochondrial Dysfunction

**FIGURE 6:**
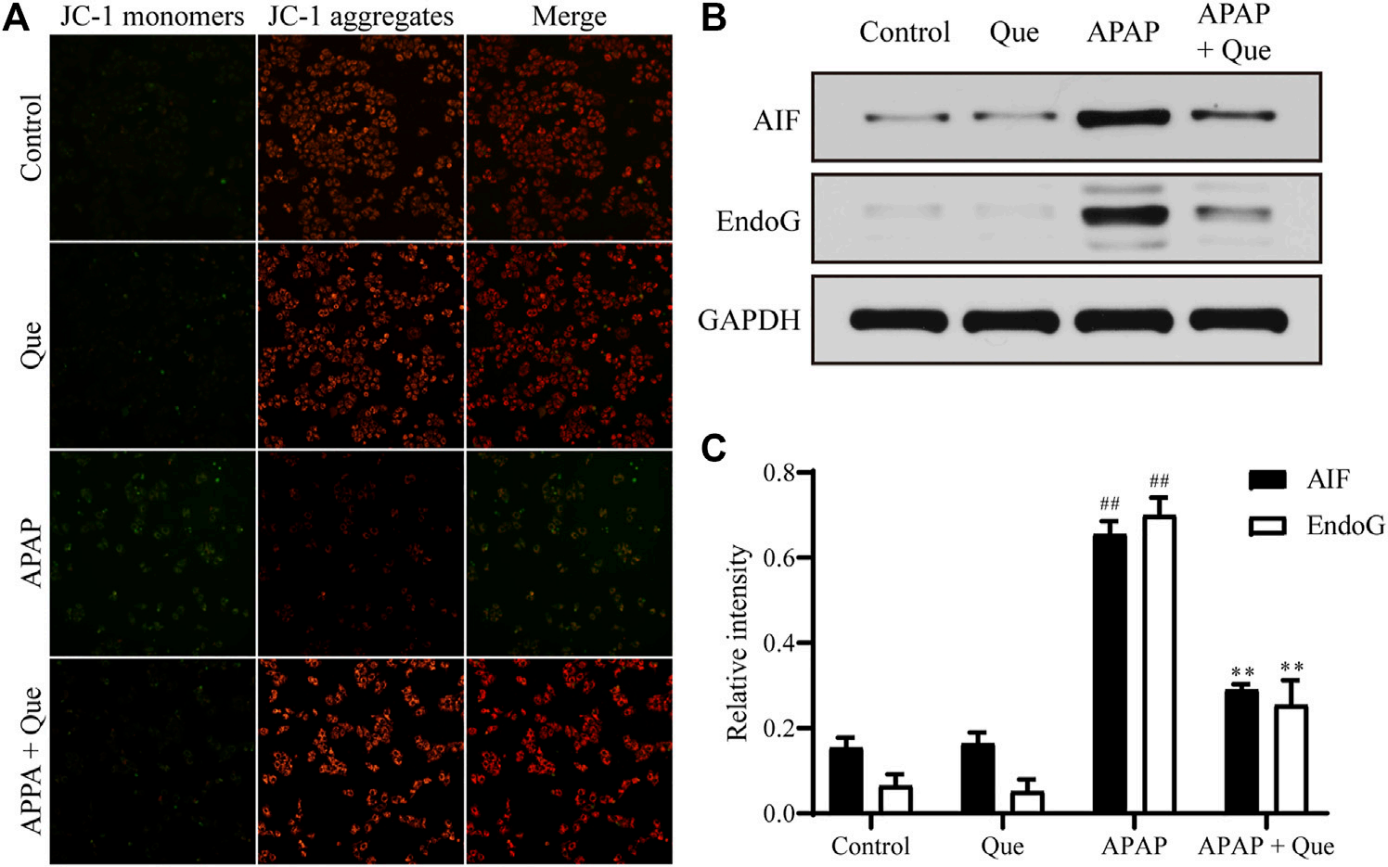
Effect of quercitrin on mitochondria. **(A)** Effect of quercitrin on mitochondrial membrane potential. **(B)** Western blot analysis of AIF and EndoG in L-02 cells. **(C)** Western blot densitometric analysis. The experimental data are expressed as mean ± SD, and the experiment was repeated three times, ^##^ *p* < 0.01, compared with the control group; **p < 0.01, compared with the APAP group.

Mitochondrial damage leads to depolarization, and the fluorescence of the JC-1 changes from red to green. Due to the decrease in mitochondrial membrane potential, JC-1 aggregates (red fluorescence) decreased and monomers (green fluorescence) increased in the APAP group. Pretreatment with quercitrin resulted in a significant increase in red fluorescence, indicating that mitochondrial depolarization was prevented (Figure 6A). In addition, the expression of apoptosis-related proteins AIF and EndoG in the APAP group increased significantly compared with the control group (*p* < 0.01), while the expression of proteins in the APAP + Que group decreased significantly compared with the APAP group (*p* < 0.01) (Figures 6B,C). The above results showed that quercitrin can prevent mitochondrial dysfunction caused by APAP.

### Quercitrin Maintains Complex I Activity

**FIGURE 7:**
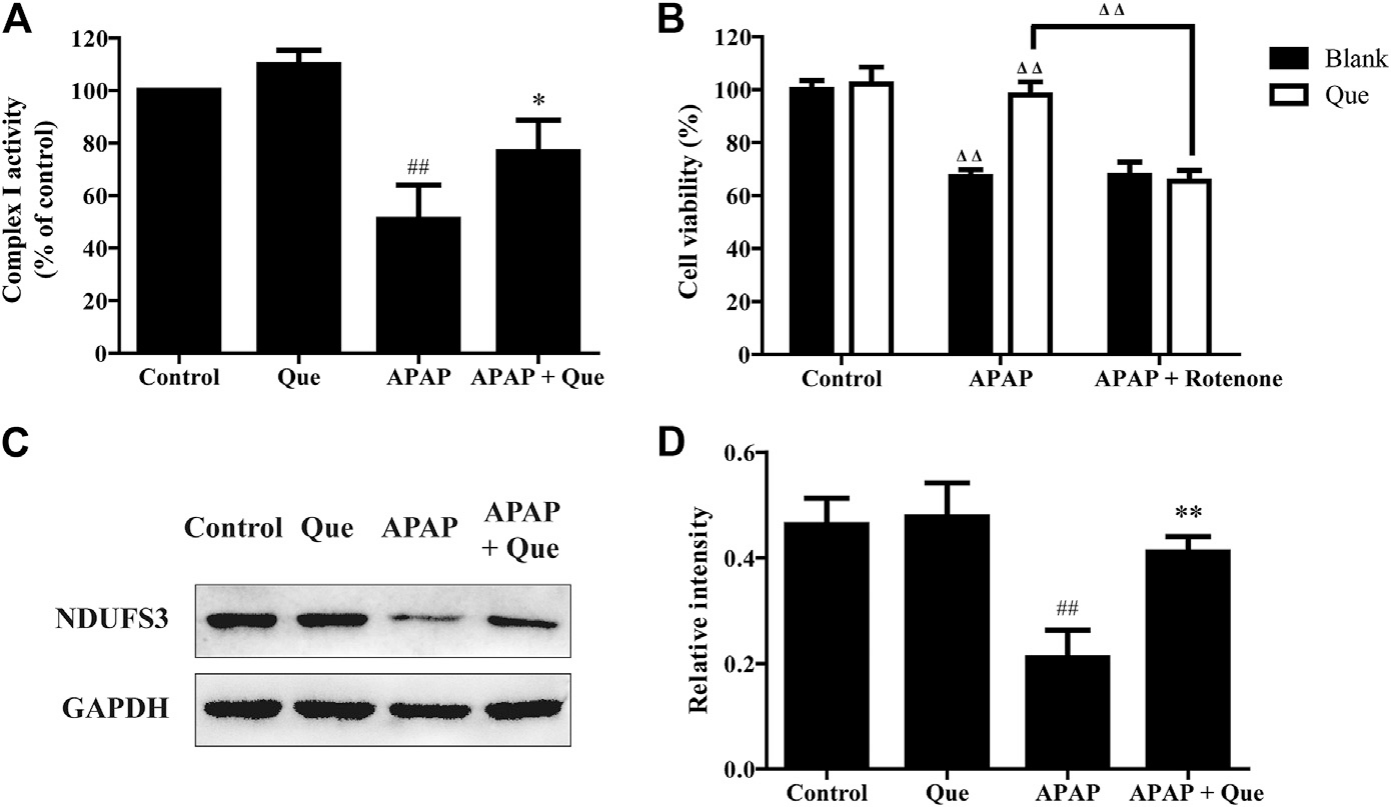
Effect of quercitrin on mitochondrial complex I. **(A)** Effect of quercitrin on the activity of complex I in L-02 cells. **(B)** Effect of APAP and rotenone treatment on L-02 cells viability in the blank group and Que group. **(C)** Western blot analysis of NDUFS3 (complex I) in L-02 cells. **(D)** Western blot densitometric analysis. The experimental data are expressed as mean ± SD, and the experiment was repeated three times, ^##^ *p* < 0.01, compared with the control group; *p < 0.05, **p < 0.01, compared with the APAP group; ^ΔΔ^ *P* < 0.01, the APAP-treated blank group was compared with the control group, the Que group was compared with the blank group, and the APAP + rotenone group was compared with the APAP group.

Although the activity of the complex I in the Que group was slightly increased when compared with the control group, there was no significant difference. The activity of the complex I in the APAP group was however significantly decreased when compared with the control group (*p* < 0.01). The complex I activity of the APAP + Que group was significantly increased when compared with the APAP group (*p* < 0.05) (Figure 7A). The results indicated that excessive levels of APAP results in a significant decrease in mitochondrial complex I activity. Pretreatment with quercitrin (80 μM) can restore the activity of complex I.

Additionally, the blank and Que groups were treated with APAP in the presence and absence of the complex I inhibitor. The smallest survival was seen in the L-02 cells in the blank group treated with APAP. Quercitrin improved the survival rate of L-02 cells treated with APAP. The combined treatment of APAP and rotenone reduced the cell survival of the Que group when compared with the Que group treated with APAP (*p* < 0.01). In contrast, the survival of the blank group treated with APAP could not be reduced further by rotenone (Figure 7B). Rotenone reduced the protective effect of quercitrin on APAP-induced cell injury by inhibiting the activity of complex I. This result indicated that the protective effect of quercitrin on the liver protection is via restoring the activity of complex I.

The expression of NDUFS3 (a well-characterized subunit of complex I) in each group was detected by Western blot. The expression of NDUFS3 (complex I) protein in the APAP group was significantly reduced when compared with the control group (*p* < 0.01). Pretreatment with quercitrin significantly increased the expression of NDUFS3 (complex I) in the APAP + Que group when compared with the APAP group (*p* < 0.01) (Figures 7C,D). The above results indicate that quercitrin maintained the activity of complex I by maintaining its protein content and thus attenuated APAP-induced cell injury.

## Discussion

Quercitrin is a flavonoid with various pharmacological activities. It is isolated from the ethyl acetate extract of Albiziae flos. We studied the mechanisms of quercitrin, which could be of a potential benefit in the attenuation APAP-induced acute liver injury.

APAP can increase the permeability of the cell membrane of the liver and released the enzymes in the cell and increased the levels of ALT, AST, and LDH in serum (Naguib et al., 2014). Quercitrin significantly reduced the levels of the abovementioned factors in the serum of mice treated with APAP and improved the pathological characteristics of the liver, indicating that quercitrin can alleviate APAP-induced liver injury in mice. The inflammatory response occurs in many pathological processes and thus becomes one of the important indicators of cell injury (Jaeschke and Ramachandran, 2020). Studies have demonstrated that oxidative stress caused by APAP leads to necrosis of hepatocytes and the release of damage-associated molecular patterns (DAMPs), including HMGB1, mtDNA, nuclear DNA, and others (Jaeschke et al., 2013). DAMPs can stimulate various inflammatory cells, mainly liver macrophages, thereby triggering pro-inflammatory transcriptional activation and the release of inflammatory mediators, such as IL-6 and TNF-α (Ramachandran et al., 2018; Wu et al., 2018; Shan and Ju, 2019). Therefore, the inflammatory response can represent the severity of APAP-induced acute liver injury. In this study, the levels of IL-6 and TNF-α in the serum of mice treated with quercitrin were decreased, indicating a lower degree of liver injury. This again suggested that quercitrin could attenuate APAP-induced acute liver injury.

ROS, including superoxide anion, singlet oxygen, hydroxyl radical, and hydrogen peroxide, can cause serious damage to nucleic acids, proteins, and membrane structures by increasing oxidative stress (Zorov et al., 2006). Many pathological oxidative damages are initially caused by ROS (Murphy, 2009). Previously published studies have demonstrated that the ROS content in the liver is significantly increased by APAP (Takemoto et al., 2014; Jiang et al., 2015; Guo et al., 2019). In addition, oxidative stress is also an important cause of APAP-induced acute liver injury (Du et al., 2016). SOD, GSH, GSH-Px, and CAT are the important enzymes for combating oxidative stress in the body (Lei et al., 2015). They protect cells and tissues from oxidative stress by directly antagonizing and neutralizing free radicals and eliminating ROS (Jodynis-Liebert et al., 2005). MDA is an imperative marker of lipid peroxidation, which is frequently used to assess the degree of tissue oxidative damage (Gaweł et al., 2004). Quercitrin reduced ROS levels in the liver of APAP-treated mice. Moreover, it also inhibited the decrease in SOD, GSH, GSH-Px, and CAT levels and the production of MDA in the liver of APAP-treated mice. These findings suggested that quercitrin decreases APAP-induced oxidative stress in mice liver cells through antioxidants.

Mitochondrial complex I is a protein complex which comprises 44 subunits (Guerrero-Castillo et al., 2017). It is also a part of the electron transport chain. Complex I is one of the important sources for the production of ROS (Onukwufor et al., 2019). The decreased activity of complex I causes the leaked electrons to directly react with oxygen or other electron acceptors, triggering a series of reactions under the action of enzymes or nonenzymes to generate various types of reactive oxygen species (Lenaz et al., 2016; Hu et al., 2017; Wang et al., 2017). MCJ protein is a chaperone molecule on the inner membrane of mitochondria, which can interact with complex I, thereby interfering with the formation of the supercomplex formed by complex I. It plays a negative role in the regulation of the respiratory chain (Genova and Lenaz, 2014). A recently published study demonstrated that the excess metabolites produced by APAP can increase the level of MCJ in the liver. This causes the interaction of MCJ with the complex, thereby interfering with the formation of the supercomplex in mitochondria, and leads to reduced ATP production and increased ROS production, which ultimately results in injury to the liver (Barbier-Torres et al., 2017). Thus, restoring the activity of complex I may be an effective treatment for APAP-induced acute liver injury.

A study on the mechanism showed that quercitrin pretreatment significantly inhibited the increase in ROS in L-02 cells induced by APAP. Furthermore, quercitrin also restored the mitochondrial membrane potential and reduced the expression of apoptosis-related proteins AIF and EndoG, which are related to mitochondrial damage (Kapoor et al., 2013). This suggested that quercitrin may prevent mitochondrial dysfunction by regulating the activity of complex I. Complex I activity assays indicated that quercitrin pretreatment restored complex I activity in injured cells. In addition, the presence of rotenone, which is a complex I inhibitor, inhibited the protective effect of quercitrin. This result further confirmed that quercitrin could protect cells by maintaining the activity of complex I and reducing the production of ROS in cells. Finally, the Western blot results demonstrated that quercitrin maintained the expression of complex I in L-02 cells which were treated with APAP. The above results indicate that the maintenance of complex I activity plays a role in quercitrin alleviating APAP-induced liver injury.

In conclusion, our results indicate that quercitrin maintains the activity of complex I by maintaining the expression of complex I, thereby reducing ROS production in mitochondria to prevent oxidative stress and ultimately reduces the cell injury induced by APAP, which indicates that quercitrin has potential value in further clinical applications.

## Data Availability

The raw data supporting the conclusions of this article will be made available by the authors, without undue reservation, to any qualified researcher.
