# Supplementary material for: Quercitrin Attenuates Acetaminophen-Induced Acute Liver Injury by Maintaining Mitochondrial Complex I Activity
Source: Front Pharmacol. 2021 May 5;12:586010. doi: 10.3389/fphar.2021.586010 (PMC8131832; doi:10.3389/fphar.2021.586010)
Supplement: Supplementary file 1 [file DataSheet1.docx]

**Supplementary data:**

**Quercitrin Attenuates Acetaminophen-Induced Acute Liver Injury by Maintaining Mitochondrial Complex I Activity**

**Weichen Xiong^1†^, Zixin Yuan^1†^, Tianshun Wang^1^, Songtao Wu^1^, Yiyi Xiong^1^, Yunfeng Yao^1^, Yanfang Yang^1,2,3,4*^ and Hezhen Wu^1,2,3,4*^**

^1^Faculty of Pharmacy, Hubei University of Chinese Medicine, Wuhan 430065, China

^2^Key Laboratory of Traditional Chinese Medicine Resources and Chemistry of Hubei Province, Wuhan 430061, China

^3^Collaborative Innovation Center of Traditional Chinese Medicine of New Products for Geriatrics Hubei Province, Wuhan 430065, China

^4^Key Laboratory of Traditional Chinese Medicine Resource and Compound Preparation Ministry of Education, Hubei University of Chinese Medicine, Wuhan 430065, China


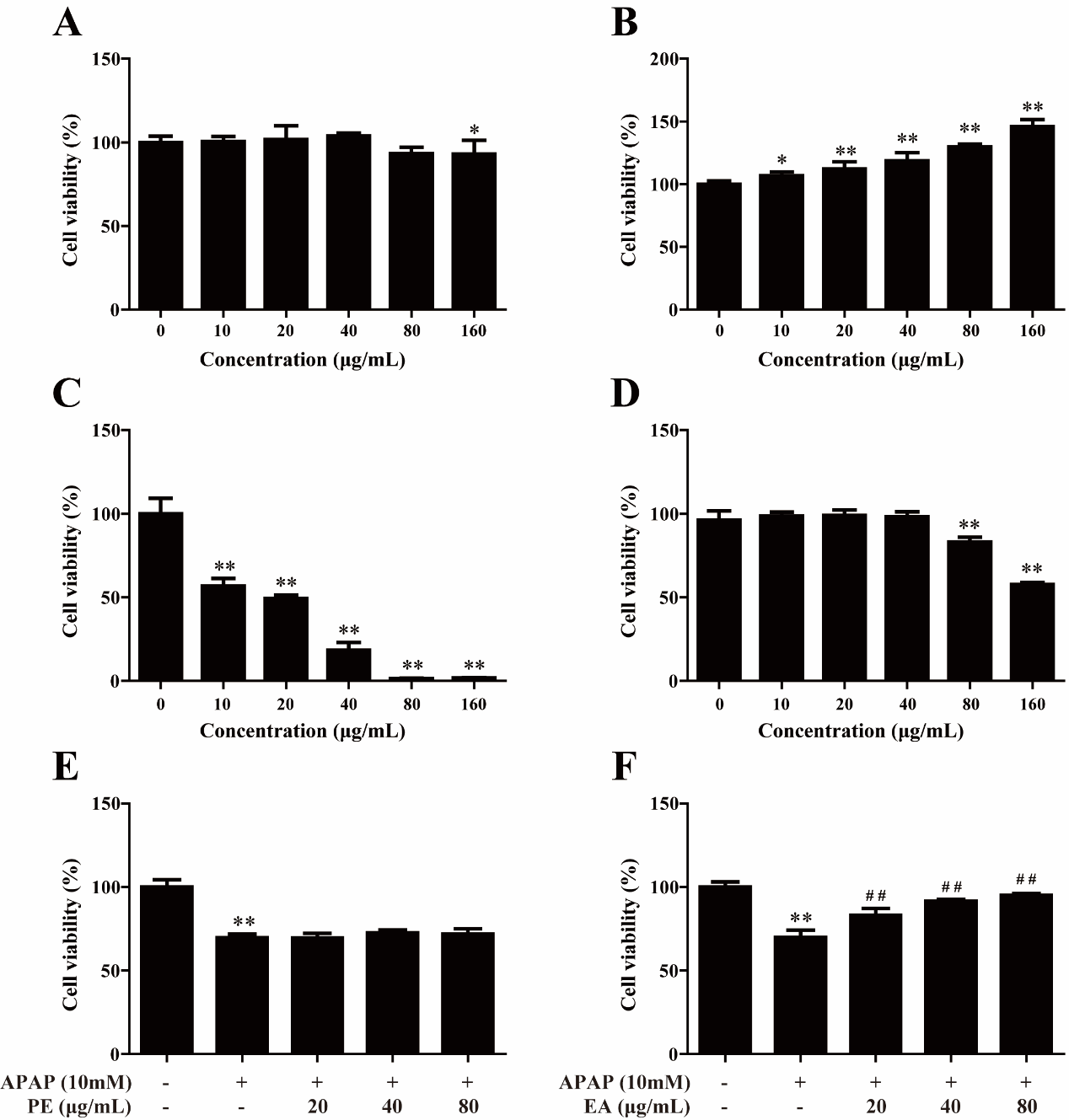


**Figure S1.** The effect of different extracts of Albiziae flos on L-02 cell activity and on L-02 cell injury induced by APAP. Effects of petroleum ether extract **(A)**, ethyl acetate extract **(B)**, n-butanol extract **(C)** and water extract **(D)** on the viability of L-02 cells. Protective effect of petroleum ether extract **(E)** and ethyl acetate extract **(F)** on L-02 cell injury induced by APAP. The experimental data were expressed as mean ± SD, **P* <0.05, ***P* <0.01, compared with the blank group; ^##^*P* <0.01, compared with the control group.


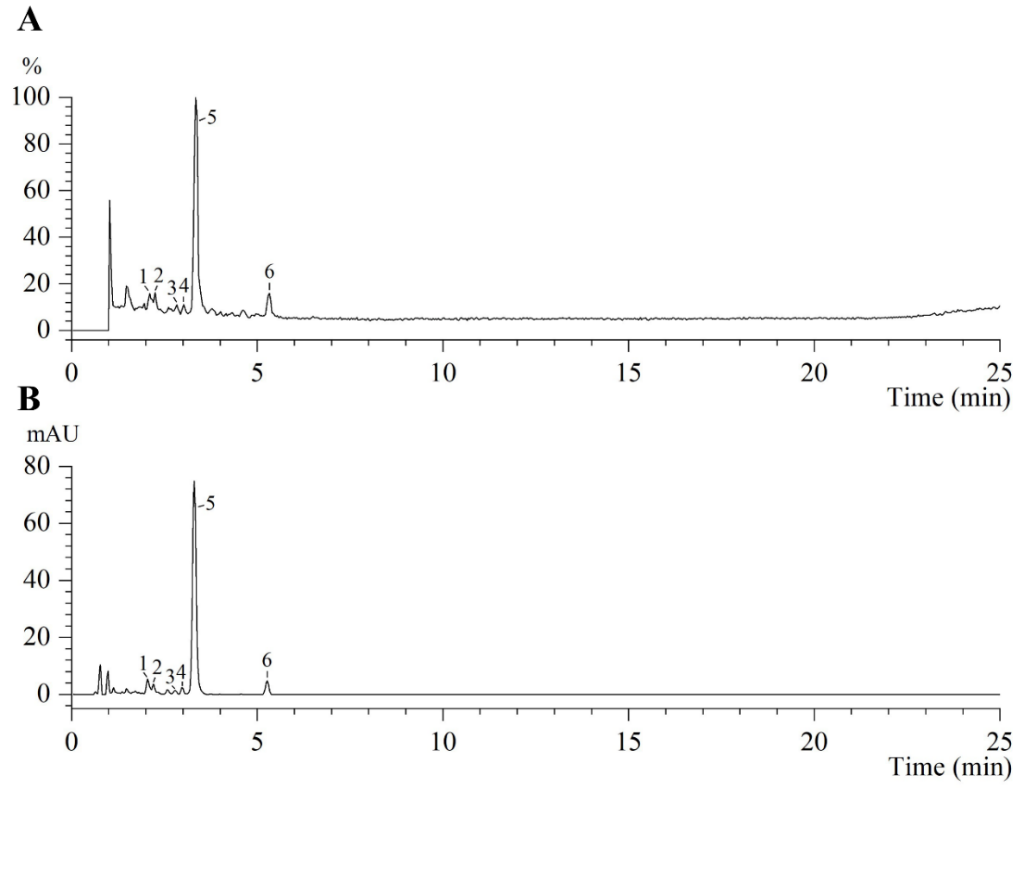


**Figure S2.** LC-MS chromatogram of the ethyl acetate extract of Albiziae Flos. **(A)** Total ion chromatogram detected in the positive ion mode. **(B)** HPLC-UV chromatogram obtained at 210 nm.

**Table S1.** Ethyl acetate extract compounds information

| No. | Time  (min) | Name | Molecular formula | [M + H]^+^ | Calcd. | [M + Na]^+^ | Calcd. |
| --- | --- | --- | --- | --- | --- | --- | --- |
| 1 | 2.10 | Myricitrin | C_21_H_20_O_12_ | 465.1025 | 465.1033 | 487.0862 | 487.0852 |
| 2 | 2.25 | Isoquecitrin | C_21_H_20_O_12_ | 465.1025 | 465.1033 | 487.0862 | 487.0852 |
| 3 | 2.84 | Luteoloside | C_21_H_20_O_11_ | 449.1039 | 449.1084 | 471.0881 | 471.0903 |
| 4 | 3.02 | Guajavarin | C_20_H_18_O_11_ | 435.0911 | 435.0927 | 457.0748 | 457.0747 |
| 5 | 3.34 | Quercitrin | C_21_H_20_O_11_ | 449.1083 | 449.1084 | 471.0881 | 471.0903 |
| 6 | 5.33 | Afzelin | C_21_H_20_O_10_ | 433.1128 | 433.1135 | 455.0952 | 455.0954 |

**Table S2.** ^1^H (500 MHz) and ^13^C (126 MHz) NMR data of quercitrin (MeOD, δ, ppm, J/Hz)

| **C atom** | **δ_C_** | **δ_H_** | **C atom** | **δ_C_** | **δ_H_** |
| --- | --- | --- | --- | --- | --- |
| 2 | 157.92 |  | 3' | 145.02 |  |
| 3 | 134.85 |  | 4' | 148.4 |  |
| 4 | 178.26 |  | 5' | 114.97 | 6.91 (d, J = 8.3 Hz) |
| 5 | 161.83 |  | 6' | 121.47 | 7.31 (dd, J = 8.3, 2.2) |
| 6 | 98.41 | 6.20 (d, J = 2.1) | 1'' | 102.15 | 5.35 (s) |
| 7 | 164.47 |  | 2'' | 70.64 | 3.75 (m) |
| 8 | 93.31 | 6.37 (d, J = 2.1 Hz) | 3'' | 70.72 | 4.22 (m) |
| 9 | 157.13 |  | 4'' | 71.86 | 3.34 (m) |
| 10 | 104.51 |  | 5'' | 70.51 | 3.42 (m) |
| 1' | 121.58 |  | 6'' | 16.26 | 0.94 (d, J = 6.2) |
| 2' | 115.54 | 7.34 (d, J = 2.1 Hz) |  |  |  |
